# Mental health first aid training of the public in a rural area: a cluster randomized trial [ISRCTN53887541]

**DOI:** 10.1186/1471-244X-4-33

**Published:** 2004-10-23

**Authors:** Anthony F Jorm, Betty A Kitchener, Richard O'Kearney, Keith BG Dear

**Affiliations:** 1 Centre for Mental Health Research, Australian National University, Canberra, ACT 0200, Australia; 2 School of Psychology, Australian National University, Canberra, ACT 0200, Australia

## Abstract

**Background:**

A Mental Health First Aid course has been developed which trains members of the public in how to give initial help in mental health crisis situations and to support people developing mental health problems. This course has previously been evaluated in a randomized controlled trial in a workplace setting and found to produce a number of positive effects. However, this was an efficacy trial under relatively ideal conditions. Here we report the results of an effectiveness trial in which the course is given under more typical conditions.

**Methods:**

The course was taught to members of the public in a large rural area in Australia by staff of an area health service. The 16 Local Government Areas that made up the area were grouped into pairs matched for size, geography and socio-economic level. One of each Local Government Area pair was randomised to receive immediate training while one served as a wait-list control. There were 753 participants in the trial: 416 in the 8 trained areas and 337 in the 8 control areas. Outcomes measured before the course started and 4 months after it ended were knowledge of mental disorders, confidence in providing help, actual help provided, and social distance towards people with mental disorders. The data were analysed taking account of the clustered design and using an intention-to-treat approach.

**Results:**

Training was found to produce significantly greater recognition of the disorders, increased agreement with health professionals about which interventions are likely to be helpful, decreased social distance, increased confidence in providing help to others, and an increase in help actually provided. There was no change in the number of people with mental health problems that trainees had contact with nor in the percentage advising someone to seek professional help.

**Conclusions:**

Mental Health First Aid training produces positive changes in knowledge, attitudes and behaviour when the course is given to members of the public by instructors from the local health service.

## Background

Community surveys have shown that the public in many countries have poor mental health literacy [1]. Many people cannot recognise mental disorders correctly, they differ from mental health professionals in their beliefs about causes and the most effective treatments, and they have stigmatizing attitudes which hinder recognition and appropriate help-seeking. This lack of mental health literacy limits the uptake of evidence-based treatments and leads to lack of support for people with mental disorders from others in the community.

To help improve mental health literacy, a Mental Health First Aid training course has been developed. This course uses the first aid model that has been successfully applied to training members of the public to help in accidents and emergencies [2]. The Mental Health First Aid course is designed to give skills to provide initial help in mental health crisis situations and for on-going mental health problems. The course teaches a five-step approach to first aid: 1. Assess risk of suicide or harm, 2. Listen non-judgmentally, 3. Give reassurance and information, 4. Encourage person to get appropriate professional help, and 5. Encourage self-help strategies. These steps are applied to depression, anxiety disorders, psychosis and substance use disorders. In addition, participants are given specific instruction on how to help in the following mental health crisis situations: a suicidal person, a person having a panic attack, a person who has experienced a traumatic event, and a psychotic person threatening violence.

An initial uncontrolled evaluation of the course involved comparing the first 210 participants at the beginning and end of the course, and at 6 months follow-up [3]. The course was found to produce improvement in ability to recognize a mental disorder in a case vignette, to change beliefs about treatment to be more like those of health professionals, to decrease social distance from people with mental disorders, to improve confidence in providing help to others, and to increase the amount of help actually provided.

The next stage in the evaluation of Mental Health First Aid involved a randomized controlled trial with 301 employees of two large government departments [4]. Participants were assigned to either receive the course immediately or were placed on a wait-list for 5 months and received the training after the trial was completed. The trial found a number of benefits, including greater confidence in providing help to others, greater likelihood of advising people to seek professional help, improved concordance with health professionals about treatments, and decreased social distance from people with mental disorders. A surprising finding was that the course improved the mental health of the participants themselves, even though they were not recruited to have mental health problems and no therapeutic benefit was promised. The mental health benefits of the course had not been assessed in the earlier uncontrolled trial.

This study involved an "efficacy" trial in that it was carried out under fairly ideal conditions which permitted rigorous experimental control. There was only one instructor who was the originator of the Mental Health First Aid course and very experienced, the trial was carried out in a workplace setting where employees were allowed time off to participate, the participants were a relatively well educated group of civil servants, and it was possible to randomly allocate participants relatively easily. In order to evaluate the course under more typical circumstances, we have now carried out a second trial. This "effectiveness" trial involved members of the public in a large rural area of Australia, who were taught by trained Mental Health First Aid instructors from the local health service. As in the previous trial, participants who received training were compared to a wait-list control group. Participants were randomized by Local Government Area clusters rather than individually because (1) there might have been contamination of information provided across allocated groups (2) the wait list group might have been difficult to maintain if others in the same locality were seen to be receiving training, and (3) individual randomization in some small communities may not have produced sufficient numbers to run a course.

The reason for basing the trial in a rural area is that people living in rural Australia are less likely to receive general practitioner services for common mental disorders and also have more limited access to specialist mental health services [5,6]. There is therefore a greater need to develop community capacity to support those with mental disorders.

## Methods

The details of this trial have been reported according to the CONSORT statement for cluster randomized trials [7].

### Participants

Eligible participants were residents of the catchment area of the New South Wales (Australia) Southern Area Health Service who were over 17 years of age, who volunteered for training in response to publicity, who were available over the period of the trial, and who were willing to receive interviews assessing trial outcomes. Participants had to volunteer as individuals rather than as a group (e.g. a whole workplace). Publicity took the form of talks to community groups, newspaper ads, a press release and radio interviews.

Eligible clusters were the 16 Local Government Areas (cities or shires) in the catchment area of the Southern Area Health Service in 2003. This catchment is located in south-east New South Wales, runs approximately 370 km from north to south and approximately 160 km from east to west, and had a population of 194,435 in 2001. The Local Government Areas varied from popular coastal areas to farming communities to rural towns and ranged in population size from less than 5000 to over 50,000.

### Intervention

**Figure 1:**
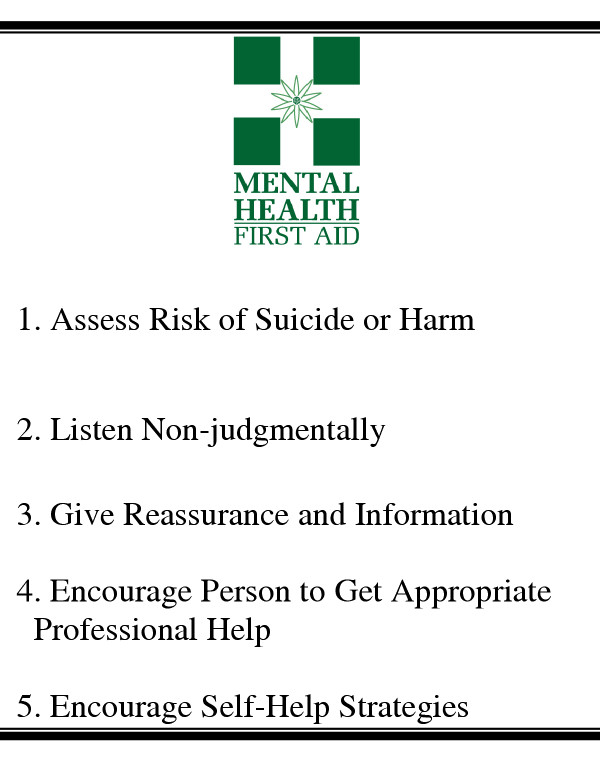
The five steps in providing mental health first aid.

Participants received a nine-hour Mental Health First Aid course, in three weekly sessions of three hours each. Training was administered in the local area in groups of up to 25 participants, with a minimum of 10 participants per course. As documentation of the intervention, there is a lesson plan for each session and a participants' manual containing material that was given to take away [2]. All instructors were given training and a teaching kit of lesson plans, videos, books, master copies of handouts and a set of transparencies. Educators received a one-week training program in how to conduct Mental Health First Aid courses and subsequent supervision in running a course. They were trained by Betty Kitchener who devised the Mental Health First Aid course. The course teaches how to help people in the crisis situations of being suicidal, having a panic attack, being exposed to a traumatic event, or in an acute psychotic state. The symptoms, risk factors and evidenced-based treatments (medical, psychological, alternative and self-help) for the mental disorders of anxiety, depressive and substance use and psychotic disorders are also taught. Figure 1 shows the five steps of providing mental health first aid taught in the course. Participants received training either immediately (experimental Local Government Areas) or after 6 months on a wait-list (control Local Government Areas).

Training was administered by educators who were recruited from the staff of the Southern Area Health Service. Expressions of interest to become Mental Health First Aid instructors were sought from staff of the Area Health Service and associated community organisations. Five Mental Health first Aid instructors were recruited from a pool of 10 applicants for these positions. All the instructors had experience in mental health work and also a background in training, working with communities or health promotion work. A project coordinator with experience in mental health and health promotion (Ms Karen Peterson), who was employed to work on the project half time, also trained as an instructor. The same instructors taught courses in each paired Local Government Area, so that this factor did not differ between the immediate and wait-list Local Government Areas. The coordinator monitored a sample of courses taught during the trial to assess fidelity to the lesson plans. A fidelity checklist of topics that had to be covered was developed for each session. Four of the instructors had all three course sessions checked, while one of the instructors only had two sessions checked. The percentage of topics covered correctly was 100% for four of the instructors and 81% for one of the instructors.

### Objectives

The hypotheses were that individuals trained in Mental Health First Aid, when compared to wait-list controls, would have increased knowledge of mental disorders and their treatments, decreased social distance, increased confidence in providing help, and that they would provide greater help to people experiencing mental health problems.

### Outcomes

Outcomes were measured in January–February of 2003 (the pre-test assessment), the courses were run for the intervention group in March–April of 2003, and outcomes were measured again in July–August 2003 (the follow-up assessment). The wait-list control group received courses in September–October 2003, after the follow-up assessment was completed.

All outcomes were measured at the individual level by telephone interview. The interview content was based on the questionnaire used in the uncontrolled trial of Mental Health First Aid [3]. The pre-test interview covered the following: whether the participant had ever experienced a mental health problem (yes/no), whether a family member had ever experienced a mental health problem (yes/no), the participant's confidence in helping someone (five-point scale from *1. not at all *to *5. extremely*), contact in the last six months with anyone with a mental health problem (yes/no), how many people, whether any help offered (yes/no), what type of help (open-ended question), recognition of the problem in a case vignette (randomly assigned to be a case of depression or one of schizophrenia), what participant would do to help if they knew the person in the vignette (this "mental health first aid intention" involved the presence or absence of 8 elements, arrived at by a qualitative analysis of a sample of the responses, and added up to give a scorefrom 0–8), ratings of the likely helpfulness of a range of interventions for the person in the vignette (scored to give a scale of percentage agreement with mental health professionals about treatment [3]), a social distance scale relating to the person in the vignette [8], whether the participant had had a problem like the one in the vignette, whether a family member had had a problem like the one in the vignette, participant's reason for doing the course, and sociodemographic characteristics of the participant (age, gender, education, non-English speaking background, aboriginality). The follow-up questionnaire was the same as the pre-test questionnaire except that it omitted the sociodemographic questions.

All outcomes were measured by a scripted telephone interview administered by professional interviewers. In order to reduce the length of the interview, participants were individually randomly assigned to receive either a depression vignette or a schizophrenia vignette, with the same questions asked in respect to each vignette. The interviewers were provided with an ID, name and phone number of each participant and knew whether they were giving the first or second interview to the participant. While they were not told whether the participant was in the experimental or control group, information about which group they were assigned to was given at the end of the interview script. As far as was practical given the very different sizes of the Local Government Area pairs, the same interviewers interviewed participants in each pair.

### Sample size determination

For power calculations and sample size determination, a conservative assumption was made that the waitlist control group would show improvements, possibly due to increased awareness of mental health issues, of about 50% of that of the experimental group. This corresponds to effect sizes in the range 0.28–0.31 for changes on scales and in the range 0.02–0.04 for changes in identifying the correct diagnosis. Sample size estimates using nQuery Advisor software [9] indicated that a sample size of 200 participants in each of the two groups would be sufficient to detect differences with power of at least 80% in 2-sided tests at the 0.05 level. Clustering effects of individuals in 16 Local Government Areas involved design effects of unknown magnitude in the analysis. It was assumed that these would be of the order of 20%, so that a total achieved sample sizes of 250 in each group would be sufficient to detect differences with 80% power.

### Randomization: Sequence generation

Randomization to immediate participation or wait-list was at the level of Local Government Area. The Local Government Areas were matched in pairs to have similar population and social characteristics. The variables used for matching were population size, interior vs coastal location, and an index of population education/occupation. The first listed LGA of each pair was assigned to the immediate or wait-list group at random, using the Random Integers option of Random.org [10] to generate a 1 or a 2 for each pair. For LGA pairs receiving a 1, the first member of the pair received immediate training, while for those receiving a 2 it was the second member of the pair.

Each individual participant was randomly assigned a variable (values of 1 or 2) to determine which case vignette they received during their interviews. This was done using the Random Integers option of Random.org [10]. Those assigned a 1 received the interview based on a vignette of a person who is depressed and those assigned 2 received a vignette of a person with schizophrenia.

### Randomization: Allocation concealment

Allocation was on the basis of cluster. In other words, the participant's Local Government Area determined whether they received immediate or wait-list training. Participants were not informed about their allocation to immediate or wait-list training until the end of their baseline interview.

### Randomization: implementation

Local Government Areas were matched in pairs and Anthony Jorm assigned these randomly to immediate training or wait-list. Participants were not able to attend a class from outside their own Local Government Area. There was a recruitment period for all Local Government Areas which was organized by the coordinator Karen Peterson. The coordinator and the participants who were recruited were blind to the allocation of the Local Government Area during the recruitment period. Anthony Jorm revealed the allocation to Karen Peterson after the recruitment period ended. Karen Peterson then organized class times either immediately or after a waiting period, depending on the allocation of each Local Government Area in the pair.

### Randomization: Blinding (masking)

At the time of the baseline interview, the participants did not know whether they were in an immediate or wait-list Local Government Area. However, interviewers had information at the end of the interview script telling whether the participant was assigned an immediate class or had to wait. Blinding of participants was not possible at subsequent interviews. Participants knew whether or not they had received training. While interviewers were not told the allocation of the participants in subsequent interviews, this might have become obvious during the interview if participants mentioned whether or not they had done the course. Interviewers were given a scripted interview to minimize any bias in the assessment due to knowledge of allocation.

### Ethics

Ethical approval for the study was given by the Australian National University Human Research Ethics Committee and by the ethics committee of the South Western Sydney Area Health Service.

### Statistical methods

For outcomes measured on a numeric scale, the change from pre-test to follow-up was analysed using linear regression. For binary outcomes, individuals scoring the same at pre-test and at follow-up were not used, and for those who changed, the direction of change was analysed as a binary outcome using logistic regression. Standard errors and p-values were adjusted for the cluster design using the Huber-White "sandwich" variance estimator, treating the 16 LGAs as the clusters. Analyses were corrected for differences between the LGA pairs by including this as an 8-level fixed-effect factor in the regression models. Missing data were imputed using best-subsets regression. All analysis was done using Stata version 8.2 [11].

## Results

### Recruitment and Participant flow

**Figure 2:**
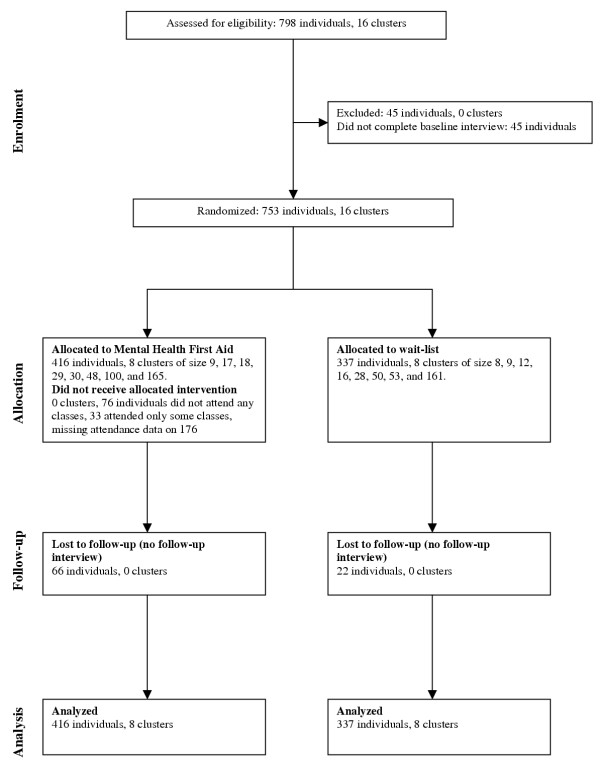
Flow diagram of the number of participants and clusters at each stage of the trial.

Recruitment of participants took place in October and November of 2002. Figure 2 shows the number of participants and clusters at each stage of the trial.

### Baseline data

Table 1 shows the characteristics of each group at the cluster and individual level. The two groups appear to be well matched in terms of sociodemographic characteristics and in history of mental health problems in self and family. However, there was a significant difference in reason for doing the course, with more people in the control group doing it for work reasons.

**Table 1:** Baseline characteristics for each group given at the individual and cluster levels.

	**Mental Health First Aid group**	**Control group**	**P-value**
**Local Government Area characteristics at baseline**			
Number	8	8	
Population size:			1.0
<5,000	3	3	
5,000–9,999	2	1	
10,000–19,999	1	2	
20,000–29,999	1	0	
30,000–39,999	1	2	
Number of participants in each area (smallest to largest)	9,17,18,29,30,48,100,165	8,9,12,16,28,50,53,161	
**Individual participant characteristics at baseline**			
Number	416	337	
Mean age (years)	47.14	47.97	0.42
Number (%) men	79 (19.0)	57 (16.9)	0.40
Number (%) with university degree	85 (20.6)	81 (24.1)	0.36
Number (%) aboriginal	11 (2.6)	10 (3.0)	0.40
Number (%) non-English speaking background	5 (1.2)	7 (2.1)	0.12
Reason for doing course:			0.011
Relating to workplace/voluntary work	180 (43.3)	188 (55.8)	
Relating to family/close friends	56 (13.5)	29 (8.6)	
Relating to own mental health status	20 (4.8)	10 (3.0)	
Duty as a citizen	49 (11.8)	44 (13.1)	
Just interested	111 (26.7)	66 (19.6)	

Note: P-values are adjusted for clustering by Local Government Area

### Numbers analyzed

The data were analyzed by an intention-to-treat approach, with single imputation used for missing data. As shown in Figure 2, the number of participants analyzed was the same as the number randomly allocated.

### Outcomes and estimation

Tables 2 and 3 show the changes found for the dichotomous and continuous outcome measures respectively and the P-value of the comparison between the Mental Health First Aid and control group on these changes. From pre-test to follow-up a significantly larger percentage of the Mental Health First Aid group than the control group changed from not reporting experiencing a mental health problem to reporting experiencing one, from incorrectly to correctly diagnosing the case vignette and from reporting not offering help to a person with a mental health problem to reporting offering help. The Mental Health First Aid group changed significantly more than the control group in their agreement with health professional about treatment, in the degree of reduction in reported social distance from the person in the vignette and in their confidence in providing help.

**Table 2:** Changes in dichotomous outcome measures.

**Outcome**	**Mental Health First Aid group**	**Control group**	**OR (95% CI) P-value **
Mental health problems in self			
Pre-test	154 (37%)	118 (35%)	
Follow-up	172 (41%)	118 (35%)	
Change (95% CI)	4% (2 to 6)	0% (-3 to 3)	0.548 (0.304, 0.986), P = 0.045
Mental health problems in family			
Pre-test	233 (56%)	183 (54%)	
Follow-up	277 (67%)	205 (61%)	
Change (95% CI)	11% (4 to 17)	7% (2 to 11)	0.575 (0.318, 1.037), P = 0.066
Correct diagnosis of vignette			
Pre-test	282 (68%)	249 (74%)	
Follow-up	337 (81%)	255 (76%)	
Change (95% CI)	13% (8 to 19)	2% (0 to 4)	0.311 (0.250, 0.387), P < 0.001
Help offered to person with mental health problem			
Pre-test	305 (73%)	256 (76%)	
Follow-up	340 (82%)	270 (80%)	
Change (95% CI)	8% (4 to 13)	4% (-2 to 10)	0.602 (0.380, 0.953), P = 0.031
Professional help advised to person with mental health problem			
Pre-test	81 (19%)	71 (21%)	
Follow-up	104 (25%)	73 (22%)	
Change (95% CI)	6% (3 to 8)	1% (-4 to 5)	0.734 (0.452, 1.191), P = 0.21

Note: P-values and confidence intervals are adjusted for clustering by Local Government Area

**Table 3:** Changes in continuous outcome measures.

**Outcome**	**Mental Health First Aid group**	**Control group**	**Treatment effect (95% CI), P-value**
Agreement with health professionals about treatment			
Pre-test mean (SEM)	60.55 (3.89)	69.46 (2.18)	
Follow-up mean (SEM)	74.74 (1.91)	70.81 (2.27)	
Change (95% CI)	14.19 (9.53 to 18.85)	1.35 (-6.04 to 8.75)	11.77 (5.98, 17.56), P = 0.001
Social distance			
Pre-test mean (SEM)	8.13 (0.24)	8.06 (0.13)	
Follow-up mean (SEM)	7.59 (0.17)	7.90 (0.20)	
Change (95% CI)	-0.53 (-0.99 to -0.08)	-0.17 (-0.41 to 0.07)	-0.26 (-0.49, -0.03), P = 0.032
Mental health first aid intention			
Pre-test mean (SEM)	1.81 (0.04)	1.88 (0.04)	
Follow-up mean (SEM)	1.83 (0.03)	1.85 (0.07)	
Change (95% CI)	0.02 (-0.11 to 0.15)	-0.03 (-0.15 to 0.08)	0.06 (-0.00, 0.12), P = 0.066
Confidence in providing help			
Pre-test mean (SEM)	3.13 (0.08)	3.17 (0.07)	
Follow-up mean (SEM)	3.39 (0.05)	3.21 (0.07)	
Change (95% CI)	0.27 (0.11 to 0.42)	0.04 (-0.02 to 0.11)	0.21 (0.10, 0.33) P = 0.001
Number of people in contact with who had mental health problem			
Pre-test mean (SEM)	3.97 (0.31)	4.56 (0.20)	
Follow-up mean (SEM)	3.89 (0.30)	4.34 (0.29)	
Change (95% CI)	-0.08 (-0.64 to 0.49)	-0.22 (-0.83 to 0.40)	0.22 (-0.18, 0.63) P = 0.25

Note: Standard errors of the mean (SEM), confidence intervals and P-values are adjusted for clustering by Local Government Area

The intraclass correlations for the continuous outcomes were: for agreement with health professionals about treatment, 0.15 (95% confidence interval 0.01, 0.29); for number of people in contact with that had a mental health problem, 0.02 (0, 0.06); for confidence in providing help, 0.03 (0, 0.07); for mental health first aid intention, 0.002 (0, 0.02); and for social distance, 0.04 (0, 0.08). Thus for all but one outcome, the correlation was small, justifying our assumption of a modest design effect.

### Adverse events

Given that an educational intervention was evaluated with a non-clinical sample, there was no justification for a systematic inquiry into adverse events. Informally, no adverse events were reported.

## Discussion

This study has found that the Mental Health First Aid training produced a number of significant changes in participants compared to a wait-list control group. A number of changes related to how people responded to a vignette of a person with either depression or schizophrenia. We found that there was greater recognition of the disorders in a vignettes, increased agreement with health professionals about which interventions are likely to be helpful, decreased social distance towards the people portrayed in the vignettes. These changes were seen equally with both vignettes. There was also a non-significant trend for those in the trained group to have more ideas for how to help the person in the vignette if it had been someone they knew.

Other outcomes with significant changes related more directly to the provision of mental health first aid. There was increased confidence in providing help to others and an increase in help actually provided. There was no change in the number of people with mental health problems that trainees had contact with or in the percentage advising someone to seek professional help.

One potential concern of Mental Health First Aid training is that it will lead to over-diagnosis of life problems as mental disorders. In previous trials we have found no evidence that the training affects the perception that the participant or their family have mental health problems [3,4]. By contrast, in the present study there was a significant increase in the percentage who perceived themselves as having a mental health problem and a non-significant trend for an increased perception of family members as having mental health problems. However, in absolute terms the changes were not so great as to be a concern and may, in fact, reflect accurate re-labelling.

These findings are similar to those of the earlier efficacy trial. However, the courses were taught by instructors who were not the originators of the Mental Health First Aid program under conditions which more closely approximate those that are typical in practice. The findings are therefore more generalizable than those reported previously.

While the more typical conditions of this trial are an advantage for generalizability, they produced greater practical difficulties in running the trial. An important weakness was that attendance data on participants were not collected by some of the instructors. We are therefore uncertain what proportion of the participants received the complete training course. A similar problem was determining the adherence of the instructors to the curriculum. We were able to carry out some formal observation of the instructors'adherence to a list of topics covered by the curriculum and found 100% adherence for most of the instructors, but one had only 81% adherence.

Another limitation of this study is that we did not directly measure the mental health of participants. In the earlier trial, we unexpectedly found a mental health benefit and this requires replication. The reason that a mental health measure was not included was that we did not have the results of the earlier trial at the time we designed this one. Another factor was the limited time available in the telephone interviews used to assess outcomes.

We used an intention-to-treat approach to the data. Whereas many trials use a last observation carried forward approach to handle missing post-test data, we used data imputation by best-subsets regression. This approach is likely to give better estimates than conventional approaches to missing data even when the missing-at-random assumption is not met [12].

Since this and the earlier trials were started, the Mental Health First Aid course has been extended from 9 to 12 hours on the basis of consistent requests from trainees for a longer course. The longer course does not add new content, but rather extends the time available to deal with each topic. We have yet to evaluate whether this extension adds to the effectiveness of the training.

## Conclusions

A nine-hour Mental Health First Aid training produces positive changes in knowledge, attitudes and behavior when the course is given to members of the public by instructors from the local health service. This finding shows that the effects of the course are generalizable beyond its originators and when run under typical conditions.

## Competing interests

BAK and AFJ were the developers of the Mental Health First Aid course.

## Authors' contributions

AFJ was involved in securing funding for the study, had a major role in the design of the study, co-developed the evaluation questionnaire, contributed to the data analysis and had a major role in writing the manuscript.

BAK was involved in securing funding for the study, developed and taught the Mental Health First Aid Instructor course, had a role in the design of the study, co-developed the evaluation questionnaire, organized the outcome assessment and had a minor role in writing the manuscript.

ROK was involved in securing funding for the study, had a role in the design of the study, had a major role in planning and managing the trial's implementation in its initial stages, recruited and supervised the study staff, established and maintained organisational support in the Southern Area, and had a role in the writing of the manuscript.

KBGD had a major role in the data analysis and a minor role in writing the manuscript.

All authors read and approved the final manuscript.

## Pre-publication history

The pre-publication history for this paper can be accessed here:



## References

[B1] Jorm AF (2000). Mental health literacy: public knowledge and beliefs about mental disorders. Br J Psychiatry.

[B2] Kitchener BA, Jorm AF (2002). Mental Health First Aid Manual.

[B3] Kitchener BA, Jorm AF (2002). Mental health first aid training for the public: evaluation of effects on knowledge, attitudes and helping behavior. BMC Psychiatry.

[B4] Kitchener BA, Jorm AF (2004). Mental health first aid training in a workplace setting: A randomized controlled trial [ISRCTN13249129]. BMC Psychiatry.

[B5] Caldwell TM, Jorm AF, Knox S, Braddock D, Dear KBG, Britt H (2004). General practice encounters for psychological problems in rural, remote and metropolitan areas in Australia. Aust N Z J Psychiatry.

[B6] Parslow RA, Jorm AF (2000). Who uses mental health services in Australia? An analysis of data from the National Survey of Mental Health and Wellbeing. Aust N Z J Psychiatry.

[B7] Campbell MK, Elbourne DR, Altman DG (2004). CONSORT statement: extension to cluster randomised trials. BMJ.

[B8] Link BG, Phelan JC, Bresnahan M, Stueve A, Pescosolido BA (1999). Public conceptions of mental illness: labels, causes, dangerousness, and social distance. Am J Public Health.

[B9] Elashoff JD (2002). nQuery Advisor – Version 50 User's Guide.

[B10] Random.org Website. http://www.random.org/.

[B11] StataCorp (2003). Intercooled Stata 82 for Windows.

[B12] Schafer JL, Graham JW (2002). Missing data: our view of the state of the art. Psychol Methods.

